# Case of Opium Poisoning

**Published:** 1876-05

**Authors:** A. R. Alley

**Affiliations:** Atlanta, Ga.


### CASE OF OPIUM POISONING.

By Dr. A. R. ALLEY, Atlanta, Ga.

I was called in consultation with Dr. W. R. D. Thompson, of this city, to see a young lady, Miss ——, aged 18 years, who had taken one and a half ounces of laudanum. Dr. Thompson arrived some twenty-five minutes after she had taken the above named quantity, and the Doctor found her almost in a moribund condition, and the symptoms of an alarming nature, with semi-comatose state; pulse about thirty per minute; contracted pupils (small pin hole) with excessive injective conjunction; stentorious breathing, with extremities cold and livid, with cutes auserina condition of skin, in which we gave sulphate zinci, grs. xx, pulv. Ipecac, grs. xl, until free emesis was established. Being impossible to procure a stomach pump, we resorted to this method, and facilitated free emesis by giving her warm water until we were confident that the stomach was thoroughly evacuated, and gave her a solution composed of sulp. atropia, grs. ij, aqua dist. z. ij ; sig. teaspoonful. Giving her teaspoonful as a dose every fifteen minutes until 1½ grains had been taken by the mouth, and the pupils of the eyes became dilated, and increase of pulse—using mustard cataplasms to wrist and thighs, along with flagilation with wet towels constantly kept up until reaction had commenced; and increase of pulse and skin became warm; whisky was then given her in free and large doses until reaction was thoroughly restored and all toxical effects of the opium subsided. This young lady was never in the habit of taking any preparation of opium, being a "love affair," she resorted to this method of committing suicide. This case presents one of the most aggravated of opium poisoning, and the large quantity taken, and some time elapsed before any medical aid was summoned, would not this be considered a case of extreme poisoning? Yet being controlled and its toxical effects corrected by the prompt and large doses of artropia. The symptoms from its commencement was of the gravest character, and which called for a quick and heroic remedy, which was administered, and a speedy recovery of the life of a young person was saved by a prompt and certain antidote to opium.

